# How do environmental protection expenditure and green technology innovation affect synergistically the financial performance of heavy polluting enterprises? Evidence from China

**DOI:** 10.1007/s11356-022-21908-1

**Published:** 2022-07-19

**Authors:** Yongjun Tang, Saifan Yue, Wenchao Ma, Lulu Zhang

**Affiliations:** 1 Business School, Hohai University, Nanjing, 211100 China; 2 School of Accounting, Zhejiang Gongshang University, Hangzhou, 310018 China

**Keywords:** Environmental protection expenditure, Green technology innovation, Financial performance, Synergetic effect, Lag effect, China

## Abstract

In recent years, economic growth has caused an increasing number of environmental problems in China. In order to achieve the goal of carbon peak on schedule, enterprises need to accelerate green transformation and upgrading. Environmental protection expenditure and green technology innovation are important means of corporate environmental governance strategy, but it is unknown whether they can promote the sustainable development of enterprises. Therefore, this article will analyze the effect of enterprise environmental protection expenditure and green technology innovation on financial performance. Based on relevant theories, this study builds a theoretical model to demonstrate how enterprise environmental protection expenditure and green technology innovation can affect the financial performance of heavy polluting enterprises. Empirical tests are carried out using 293 heavy polluting enterprises in China as the sample. The results reveal that: (i) Enterprise environmental protection expenditure has significant negative effects on current enterprise financial performance, while green technology innovation can significantly promote enterprise financial performance. (ii) When the lag period is two periods, the enterprise environmental protection expenditure and green technology innovation have positive effects on enterprise financial performance respectively, and the effects are the most significant. (iii) Enterprise environmental protection expenditure and green technology innovation synergistically promote enterprise financial performance in the current period, and the impact has a lag effect. (iv) In state-owned enterprises and enterprises with higher corporate governance level, the synergetic promotion effect of environmental protection expenditure and green technology innovation on enterprise financial performance is more significant. Finally, this study provides suggestions for promoting the transformation and upgrading of heavy polluting enterprises and achieving sustainable development from the perspectives of the government, enterprises and the public.

## Introduction

Enterprise environmental protection expenditure and green technology innovation are important issues within the topic of sustainable development because they significantly influence ecological protection and environmental governance (Li et al. 2021; Liu et al. 2022; Cheng et al. 2022; Wang et al. 2022a). Since the reform and opening up, with the rapid development of China’s economy, various environmental problems have emerged, which has gradually become a major obstacle to the realization of green and sustainable development in China (Zhou and Li 2021; Tao et al. 2021). The “environmental pollution–economic development” circle is a problem in the process of national sustainable development (Guo et al. 2018). In this context, urging enterprises to increase expenditure on environmental protection projects and green technology innovation to realize industrial transformation and upgrading has become a new direction of China’s economic development, and has been raised to the strategic height of national development (Du et al. 2021).

At the Fifth Plenary Session of the 18th Communist Party of China (CPC) Central Committee, the five development concepts of “innovation, coordination, green, openness and sharing” were put forward. The concept of green development was taken as an important guiding ideology for national economic development, pointing out the direction for ecological environment protection and green technology innovation. The report of the 19th CPC National Congress proposed to “build a market-oriented green technology innovation system”. In order to further implement the relevant top-level design of the CPC Central Committee, *the Guiding Opinions on Building a Market-Oriented Green Technology Innovation System* was issued in April 2019, which further refined the road map and timetable for the construction of green technology innovation system, “Green technology innovation” has become an important task in the construction of ecological civilization in China. Green technology innovation means that enterprises realize green transformation at the source of production links through the research and application of green products and green processes. Green technology innovation can reduce the emission of waste and improve the utilization of resources (Lin and Ma 2022). Green products will also bring economic benefits to enterprises. However, green technology innovation activities have the risk of uncertainty, and the R&D cost is difficult to be compensated. Environmental protection expenditure refers to the expenditure incurred to control the pollution caused by the production and operation activities of enterprises (Zhang et al. 2022). If enterprises increase expenditure in environmental protection, it will lead to an increase in the operating costs in the short term, which is not conducive to the financial performance. In the long term, enterprises’ expenditure in environmental protection is conducive to shaping the corporate image of green environmental protection, making enterprises stand out in the fierce competition and helping enterprises achieve sustainable development. Therefore, it is necessary to discuss the impact of environmental protection expenditure and green technology innovation on enterprises from the short-term and long-term perspectives.

Enterprises are one of the main subjects of resource consumption and environmental damage. The production and operation activities of Chinese enterprises lead to 80% of environmental pollution problems. According to the principle of “who develops, who protects, who pollutes, who recovers”, enterprises should bear the responsibility of environmental protection and reduce pollution emission during production. Under the background of putting the construction of ecological civilization in a prominent position proposed by the 19th National Congress of the Communist Party of China, the intensity of environmental regulation in China has reached an unprecedented level, and heavy polluting enterprises are the main regulatory objects of environmental regulation measures (Wang et al. 2022b). In order to achieve the goal of carbon peak on schedule, people from all walks of life pay more attention to the pollution discharge of heavy polluting enterprises (Yang et al. 2022a, 2022b). In recent years, many places in China have strictly implemented a series of environmental regulatory measures, such as limiting production and stopping production, improving production standards, saving energy and reducing emission and so on, which has prompted many enterprises in heavy polluting industries to increase environmental protection expenditure and carry out green technology innovation (Shang et al. 2022; Wu et al. 2022). Enterprise environmental protection expenditure and green technology innovation are “double edged swords”. On the one hand, due to increasing environmental protection expenditure and green technology innovation, enterprises have established a “green” corporate image, which makes it easier to obtain the support of stakeholders, develop green innovative products and improve their core competitiveness (Aguilera-Caracuel and Ortiz-de-Mandojana 2013). On the other hand, the application of enterprise funds in environmental protection expenditure and green technology innovation also increases the burden on enterprises and brings challenges to enterprise management (Song et al. 2017). Through the above two environmental governance behaviors, some enterprises in heavy pollution industries are heavily in debt and finally shut down; however, some enterprises seize the historical opportunity, not only realizing the transformation and upgrading of enterprises through environmental protection expenditure and green technology innovation, but also finally improving the financial performance of enterprises. Therefore, what are the reasons for the above differences among heavy polluting enterprises? How do environmental protection expenditure and green technology innovation affect the financial performance of enterprises? How to carry out environmental protection expenditure and green technology innovation to better improve the financial performance of enterprises? The research on the relationship between environmental protection expenditure, green technology innovation and financial performance may provide an answer. Existing scholars have studied the relationship between environmental protection expenditure and financial performance of enterprises and the relationship between green technology innovation and financial performance of enterprises respectively (Qing et al. 2022). The research results are becoming increasingly rich, gradually involving various specific industries, and the conclusions are different due to the different choices of empirical samples and measurement methods. There are few studies on the relationship among environmental protection expenditure, green technology innovation and financial performance. This study argues that we should not analyze the impact of environmental protection expenditure and green technology innovation on financial performance in isolation.

Based on the above analysis, this study takes China listed enterprises in the heavy polluting industry from 2015 to 2019 as the research object. The purpose of the article is to put enterprise environmental protection expenditure, green technology innovation and financial performance into a theoretical framework, systematically analyze the relationship among the three to explore their internal logical mechanism and better understand and explain the relationship among the three. On the basis of considering the lag effect, this study analyzes the relationship between enterprise environmental protection expenditure and green technology innovation of the enterprises in the heavy pollution industry and their financial performance respectively, then discusses the synergetic effect of the enterprise environmental protection expenditure and green technology innovation on financial performance. We also discuss the synergetic effect under different property rights and corporate governance levels.

This study examined the synergetic effects of enterprise environmental protection expenditure and green technology innovation on their financial performance. The contribution of this study can be summarized into three aspects: (i) There are still different conclusions on the relationship between enterprise environmental protection expenditure, green technology innovation and financial performance at home and abroad. According to the objective requirements of carbon peak, this study discusses the impact of environmental protection expenditure and green technology innovation on enterprises from the short-term and long-term perspectives, and finds the lag effect. (ii) This study discusses the relationship between enterprise environmental protection expenditure, green technology innovation and financial performance from the perspective of synergy for the first time, and studies the synergy mechanism of enterprise environmental protection expenditure and green technology innovation on enterprise performance. (iii) In this study, the principal component analysis method is used to construct the evaluation system of corporate governance level. We also discuss the effects of enterprise environmental protection expenditure and green technology innovation on financial performance under different property rights and corporate governance levels. Our research will help to fully understand the mechanism of environmental protection expenditure and green technology innovation on enterprise performance and provide suggestions for enterprises to use a variety of means to carry out environmental governance. So, this study has guiding significance for promoting enterprise green transformation and realizing sustainable development.

## Literature review and research hypothesis

### Environmental protection expenditure and financial performance of enterprises

The research on the relationship between enterprise environmental protection expenditure and financial performance has achieved rich research results, but there are great differences in the research conclusions of different scholars (Akbar et al. 2021). Theoretically, the resource-based theory holds that enterprise environmental protection expenditure has a significant negative effect on financial performance. If an enterprise chooses to carry out environmental protection project construction within limited resources, it is bound to occupy the resources of other business projects, which will increase the enterprise cost, reduce the enterprise profit margin and have a negative impact on the enterprise’s financial performance in the short term (Hassel et al. 2005). Stakeholder theory, social responsibility theory, organizational legitimacy theory and other theories believe that enterprise environmental protection expenditure has a positive effect on financial performance. Enterprises increase environmental protection expenditure and take the initiative to bear social responsibility, which can convey positive information to stakeholders and create a “green” corporate image. It is also conducive to enterprises to obtain government support, which will have a positive impact on the financial performance of enterprises (López-Gamero et al. 2010; Russo and Fouts 1997; Hart and Ahuja 1996).

From the perspective of empirical research, the first view is that enterprise environmental protection expenditure is positively correlated with financial performance. Using a cross-sectional sample of 198 US “Standard & Poors 500” firms, Al-Tuwaijri et al. (2004) find that “good” environmental performance is significantly associated with “good” economic performance. Earnhart (2007) believe that through active investment in environmental protection, enterprises can establish a green corporate image, improve the core competitiveness of products, reduce the pollutant emission and environmental tax burden of enterprises, stimulate innovation compensation, attract the attention and favor of investors, make it easier to obtain capital financing and improve the financial performance. de Burgos-Jiménez et al. (2013)used regression analysis on a combined sample of 186 Welsh companies to evaluate the effect on performance of different types of environmental protection and found a positive effect of environmental protection on mid-term financial performance. The second view is that enterprise environmental protection expenditure is negatively correlated with financial performance. Filbeck and Gorman (2004) believe that enterprise environmental protection expenditure will negatively affect the financial return of enterprises. Jaggi and Freedman (1992) believe that environmental protection expenditure is not conducive to the market value of enterprises. The third view is that the impact of enterprise environmental protection expenditure on enterprise financial performance is uncertain. Based on the environmental perspective, Guenster et al. (2011) found that corporate environmental protection expenditure has no significant impact on financial performance, whether positive or negative. In addition, some researchers have proposed that there is a U-shaped curve relationship between enterprise environmental protection expenditure and financial performance (Jin and Xu 2020). Some literatures analyze the impact of environmental protection expenditure on the later financial performance of enterprises from a cross period perspective. Based on a data set of 16,325 firm-years covering the period from 1991 through 2011, Singal (2014) found that superior environmental performance improves future financial performance.

This paper holds that enterprises need a long process from increasing environmental protection expenditure to establishing a good image, improving product competitiveness, stimulating innovation compensation, attracting investment and then creating value. In the short term, the environmental protection expenditure of enterprises will increase the cost of enterprises, reduce the profit margin of enterprises and have a negative impact on the financial performance. However, in the long term, increasing environmental protection expenditure can convey positive information to stakeholders, create a “green” corporate image and help enterprises obtain government support, which will have a positive impact on the financial performance of enterprises.

Based on the above analysis, the first hypothesis of this study is put forward:

**H1a:** There is a negative correlation between enterprise environmental protection expenditure and current financial performance;

**H1b:** The positive impact of enterprise environmental protection expenditure on financial performance has a lag effect.

### Green technology innovation and financial performance of enterprises

Green technology innovation refers to the collection of all environment-friendly innovation activities to achieve the sustainable development goal of enterprises. Scholars at home and abroad have carried out rich research on the relationship between green technology innovation and enterprise performance, but the research conclusions have not been unified. Theoretically, technological innovation theory and stakeholder theory believe that there is a positive correlation between enterprise green technological innovation and financial performance. Green technology innovation activities can reduce the negative externalities of the environment, improve production and operation efficiency, develop green innovative products required by the market, obtain “first mover advantage”, improve market competitiveness, transmit “green” signals to investors, obtain the favor and support of the government and the public, and improve the financial performance of enterprises (Amores-Salvadó et al. 2014; Chan et al. 2016).

From the perspective of empirical research, most scholars believe that enterprise green technology innovation is positively correlated with financial performance. Sharma and Vredenburg (1998) found that enterprise green technology innovation can not only reduce production costs, but also help enterprises gain a good reputation in the market, help enterprises win more market share and improve operating revenue. Using data from China’s 209 listed companies that belong to heavily polluting manufacturing industries, Xie et al. (2019) found that both green process innovation and green product innovation can improve a firm’s financial performance. Farza et al. (2021) investigated the relationship between green innovation and corporate financial performance for German HDAX companies from 2008 to 2019 and found a linear positive effect of green innovation on different financial performance measures. A few scholars believe that enterprise green technology innovation is negatively correlated with financial performance. Przychodzen et al. (2020) demonstrated that too much concentration on green innovation relative to other types of innovative activism has a negative influence on both accounting and stock market performance. In addition, some researchers believe that there is no significant association between enterprise green technology innovation and financial performance (Duque-Grisales et al. 2020). Since it takes a certain time for green technology innovation activities to be realized from the beginning to the implementation, some scholars explained the reasons why it is difficult for green innovation ability to improve enterprise financial performance in the short term, and believed that time dimension should be taken into account in the research. de Azevedo et al. (2019) examine the role of green innovation intensity on financial performance based on data from 356 multinationals firms and found that there is no significant association of green innovation’s intensity with firm financial performance in the immediate year. But the association is positive, lasts during the subsequent years and becomes expressively higher after 2 years. Based on data of energy listed firms in China from 2008 to 2017, Chen and Ma (2021) find that green investment has a significant and positive correlation with financial performance; in the third year after investment in energy conservation and emission reduction, financial performance has been significantly improved.

To sum up, this study believes that green technology innovation can improve the production efficiency of enterprises, reduce the enterprises’ costs, transmit the “green” signal to the society, obtain the support of the government and the public, and improve the market competitiveness and current financial performance of enterprises. Since green technology innovation activities include all aspects of R&D and operation, and will experience a long time. In the later stage, with the gradual implementation of enterprise green technology innovation, it will have a more significant positive effect on the financial performance of enterprises.

Based on the above analysis, the second research hypothesis of this study is put forward:

**H2a:** There is a positive correlation between enterprise green technology innovation and current financial performance;

**H2b:** The positive impact of enterprise green technology innovation on financial performance has a lag effect.

### The synergetic effect of enterprise environmental protection expenditure and green technology innovation on financial performance

Though there are many researches about enterprise environmental protection expenditure and financial performance, enterprise green technology innovation and financial performance, there is little literature on the combination of the three. Theoretically, based on the analysis of Porter hypothesis, when enterprises face environmental regulation, they will increase environmental protection expenditure, stimulate enterprises’ technological innovation activities, bring innovation compensation and “first mover advantage”, reduce enterprises’ production costs and finally improve enterprises’ financial performance.

From the perspective of empirical research, Fan and Wang (2019) took China’s Listed Coal Companies from 2012 to 2018 as the research sample and found that environmental protection investment and green technology innovation work together on the financial performance. Enterprise environmental protection expenditure can be seen as the performance of enterprises in fulfilling their social responsibility. Lioui and Sharma (2012) demonstrated that corporate environmental social responsibility (ECSR) has a negative impact on corporate financial performance, but the interaction between ECSR and R&D investment has a positive impact on corporate financial performance.

To sum up, this study considered that enterprise environmental protection expenditure in the immediate year will increase the production costs, which is not conducive to the improvement of enterprise financial performance in the current period. However, if the enterprise actively carries out green technology innovation while increasing environmental protection expenditure, on the one hand, the enterprise’s environmental protection expenditure is used to purchase environmental protection equipment and carry out energy conservation and emission reduction projects, which changes the original technological process and equipment to a certain extent and enhances the ability of green technology innovation; On the other hand, through the innovation of green technology, green products and green management, green technology innovation can not only reduce the production and operation costs of enterprises, but also improve the enterprises’ technical barriers, obtain unique competitive advantages and effectively reduce the negative impact of environmental protection expenditure on the current financial performance. That is, enterprise green technology innovation plays a positive regulatory role between enterprise environmental protection expenditure and current financial performance. Similarly, the improvement of green technology innovation also enhances the environmental protection awareness of enterprises, which is conducive to better environmental protection expenditure and create the “green” image of enterprises, so as to maintain a good cooperative relationship with stakeholders and improve market competitiveness. This “green” image also makes it easier for enterprises to communicate their initiatives to stakeholders, including investors, customers, media and regulatory authorities on the expectation that this will encourage brand loyalty, build reputation and proactively prevent onerous regulation (Bird et al. 2007; Miles et al. 2000). In conclusion, the effect of enterprise environmental protection expenditure and green technology innovation on financial performance can form a “1 + 1 > 2” synergetic effect. Environmental protection expenditure enhances the enterprise’s green technology innovation ability. With the improvement of the enterprise’s green technology innovation ability, it also enhances the enterprise’s environmental protection awareness and urges the enterprise to increase environmental protection expenditure, so that the enterprise’s environmental protection expenditure, green technology innovation and financial performance form a virtuous circle. Since it takes a certain time for the environmental protection expenditure and green technology innovation of enterprises to take effect, it is reasonable to believe that the synergetic effect of environmental protection expenditure and green technology innovation on enterprise financial performance lags behind.

Based on the above analysis, the third research hypothesis of this study is put forward:

**H3a:** Enterprise environmental protection expenditure and green technology innovation have a positive synergetic effect on the improvement of financial performance in the current period;

**H3b:** Enterprise environmental protection expenditure and green technology innovation have a positive synergetic effect on the improvement of financial performance in the long term.

**Fig. 1:**
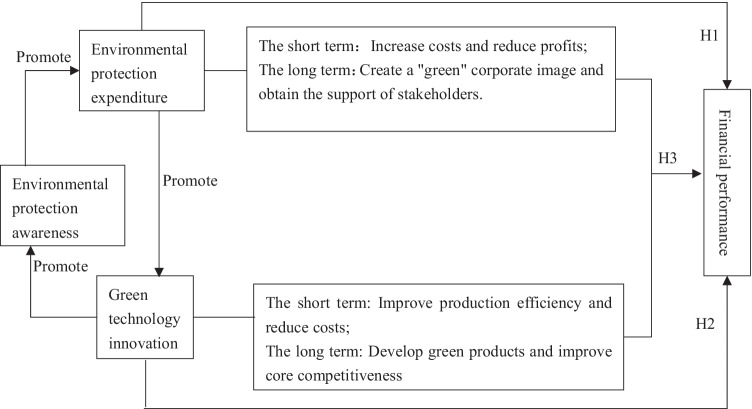
Conceptual model

According to the above analysis, the corresponding conceptual model is constructed, as shown in Fig. 1.

## Research design

### Sample selection and data source

This study selects the data of China’s A-share listed companies from 2015 to 2019 as the initial sample. According to the provisions of the guidelines for environmental information disclosure of listed companies published by the Ministry of environmental protection of China and combined with the industry classification of the China Securities Regulatory Commission (CSRC) in 2012, this study choose 16 categories of industries including thermal power, steel, cement, electrolytic aluminum, coal, metallurgy, chemical industry, petrochemical, building materials, papermaking, brewing, pharmaceutical, fermentation, textile, tanning and mining as heavy polluting industries. On this basis, this study excluded: (1) ST and * ST companies; (2) companies with serious lack of financial data. Finally, the unbalanced panel data of 293 enterprises with a sample size of 1167 were obtained. The data sources of this study are as follows: (1) manually collect the environmental protection expenditure and green technology innovation data of enterprises from the original data such as the sample company’s annual report, social responsibility report and environmental report, and manually sort them out with Excel; (2) collect other relevant data required from CSMAR database. The relevant data in this study are processed and tested by Stata 15.1.

### Variable definition

#### Dependent variable

Enterprise financial performance (ROA). In the past, there are two main methods to measure the financial performance of enterprises in the classical research literature: One is the value measurement based on the capital market, such as Tobin’s Q; the other is accounting profitability indicators, such as return on net assets and return on total assets. In most studies, scholars use profitability indicators to represent the financial performance of enterprises. Therefore, this study selects the total asset net profit margin (ROA), which can reflect the utilization efficiency of total assets, to represent the financial performance of enterprises. According to this indicator, we can judge the effect of asset utilization and the real operating state of enterprises.

#### Independent variables

Environmental protection expenditure (EPI). Based on the previous measurement methods of environmental protection investment, the ratio of total environmental protection investment to enterprise capital stock is taken to represent the level of enterprise environmental protection expenditure. Capital stock = (total assets at the beginning of the year + total assets at the end of the year) / 2. The environmental protection expenditure data of enterprises shall be counted manually in the annual report and social responsibility report of listed companies. The specific value of enterprise environmental protection expenditure comes from the deposit for environmental restoration and treatment in the “special reserve”, the sewage charge in the “management fee”, the greening fee, the investment in energy-saving and environmental protection projects in the “project under construction” and so on.

Green technology innovation (GTI). In the past, there are usually two methods to measure green technology innovation in classical research literature, one is quantitative index measurement, and the other is systematic evaluation measurement. Because this study selects heavy polluting industry enterprises as the research object, the disclosure level of green technology innovation is higher than that of other industry enterprises, so quantitative index measurement is selected. The improvement of green technology innovation needs a lot of financial support. This paper uses the ratio of green R&D to operating income to represent the green technology innovation of enterprises.

#### Control variables

According to the existing literature, this study adds a series of enterprise financial performance influencing variables to exclude the influence of other factors on the dependent variable. It mainly includes company size (SIZE), capital structure (LEV), current ratio (LIQ), equity concentration (EQU), enterprise nature (SOE) and enterprise age (AGE).

The interpretation and measurement of all model related variables in this study are shown in Table 1.

**Table 1:** Variable definition

Variable type	Variable	Symbol	Variable description
Dependent variable	Financial performance	ROA	Net profit/average balance of total assets
Independent variable	Environmental protection expenditure	EPI	Total investment in environmental protection/capital stock
	Green technology innovation	GTI	Green R&D expenditure/operating income
Control variable	Company size	SIZE	Natural logarithm of total assets at the end of the period
	Capital structure	LEV	Year-end total liabilities/year-end total assets
	Current ratio	LIQ	Year-end current assets/year-end current liabilities
	Equity concentration	EQU	Number of shares held by the largest shareholder/total number of shares
	Property rights	SOE	Dummy variable, the value of state-owned holding company is 1, otherwise it is 0
	Enterprise age	AGE	Add 1 to the company’s establishment year and then take the natural logarithm
	Year	YEAR	Set on the basis of 2015, take 1 for the current year, otherwise it is 0

### Model building

(i) In order to test the impact of enterprise environmental protection expenditure in heavy polluting industries on the current financial performance, that is hypothesis H1a, and a regression model as shown in formula (1) is specially set, where ${\alpha }_{0}$ is a constant term, *ε* is a residual term, *i* represents each listed company and *t* represents the year.

1 $${\mathrm{ROA}}_{i,t}={\alpha }_{0}+{\alpha }_{1}*{EPI}_{i,t}+{\alpha }_{2}{Controls}_{i,t}+{\varepsilon }_{i,t}$$

In order to further test the lag effect of enterprise environmental protection expenditure and financial performance in heavy polluting industries, and to verify the hypothesis H1b, and a regression model as shown in formula (2) is constructed, where *n* = 1,2.

2 $${\mathrm{ROA}}_{i,t}={\alpha }_{0}+{\alpha }_{1}*{EPI}_{i,t-n}+{\alpha }_{2}{Controls}_{i,t}+{\varepsilon }_{i,t}$$

(ii) In order to test the impact of green technology innovation of enterprises in heavy polluting industries on the current financial performance, that is hypothesis H2a, and a regression model as shown in formula (3) is specially set, where ${\beta }_{0}$ is a constant term, *ε* is a residual term, *i* represents each listed company and *t* represents the year.

3 $${\mathrm{ROA}}_{i,t}={\beta }_{0}+{\beta }_{1}*{GTI}_{i,t}+{\beta }_{2}{Controls}_{i,t}+{\varepsilon }_{i,t}$$

In order to further test the lag effect of green technology innovation and financial performance of enterprises in heavy polluting industries, and to verify the hypothesis H2b, a regression model as shown in formula (4) is constructed, where *n* = 1,2.

4 $${\mathrm{ROA}}_{i,t}={\beta }_{0}+{\beta }_{1}*{GTI}_{i,t-n}+{\beta }_{2}{Controls}_{i,t}+{\varepsilon }_{i,t}$$

(iii) In order to test whether the environmental protection expenditure and green technology innovation of enterprises in heavy polluting industries synergistically promote the improvement of the current financial performance of enterprises, and to verify the hypothesis H3a, a regression model as shown in formula (5) and (6) is specially set, where ${\gamma }_{0}$ is a constant item, *ε* is the residual item*,* i represents each listed company and *t* represents the year.

5 $${\mathrm{ROA}}_{i,t}={\gamma }_{0}+{\gamma }_{1}*{EPI}_{i,t}+{{\gamma }_{2}*{GTI}_{i,t}+\gamma }_{3}{Controls}_{i,t}+{\varepsilon }_{i,t}$$

6 $${\mathrm{ROA}}_{i,t}={\gamma }_{0}+{\gamma }_{1}*{EPI}_{i,t}+{{\gamma }_{2}*{GTI}_{i,t}+{\gamma }_{3}{EPI}_{i,t}*{GTI}_{i,t}+\gamma }_{4}{Controls}_{i,t}+{\varepsilon }_{i,t}$$

In order to further test the lag effect of environmental protection expenditure and green technology innovation of enterprises in heavy polluting industries on the financial performance of enterprises, and to verify the hypothesis H3b, a regression model as shown in formula (7) and (8) is specially set, where *n* = 1,2.

7 $${\mathrm{ROA}}_{i,t}={\gamma }_{0}+{\gamma }_{1}*{EPI}_{i,t-n}+{{\gamma }_{2}*{GTI}_{i,t-n}+\gamma }_{3}{Controls}_{i,t}+{\varepsilon }_{i,t}$$

8 $${\mathrm{ROA}}_{i,t}={\gamma }_{0}+{\gamma }_{1}*{EPI}_{i,t-n}+{{\gamma }_{2}*{GTI}_{i,t-n}+{\gamma }_{3}{EPI}_{i,t-n}*{GTI}_{i,t-n}+\gamma }_{4}{Controls}_{i,t}+{\varepsilon }_{i,t}$$

## Empirical testing and result analysis

### Descriptive analysis

It can be seen from the descriptive statistical results in Table 2: (i) The median environmental protection expenditure of the sample enterprises is 0.0028, and the average value is 0.0438, indicating that the environmental protection expenditure level of more than half of the listed companies in the heavy polluting industries in the sample is lower than the average level. Moreover, the environmental protection expenditure levels of the sample enterprises vary greatly. The environmental protection expenditure ranges from the minimum value of 0 to the maximum value of 7.8844, and the standard deviation is higher than the mean and median, indicating that the environmental protection expenditure of the sample enterprises may have a non-normal distribution; (ii) The minimum value of enterprise green technology innovation is 0, the mean value is 2.7711 and the maximum value is 8.65, indicating that there are large differences in the level of green technology innovation among sample enterprises; (iii) The overall financial performance of sample enterprises is relatively low and there are large differences, and their environmental protection expenditure and green technology innovation are in great difference, which preliminarily confirms the necessity of research; (iv) Most of the sample enterprises are large in scale, and most of the sample enterprises are state-owned enterprises, indicating that China’s heavy polluting industries are mainly controlled by the state; (v) The sample enterprises financial leverage is high, and there are greater financial risks, but at the same time, it also shows that corporate financing costs are low.

**Table 2:** Descriptive statistics

Variable	Observations	Mean	Median	Standard deviation	Min	Max
ROA	1167	0.0150	− 0.0030	0.0553	− 0.5560	0.3790
EPI	1167	0.0438	0.0028	0.3067	0	7.8444
GTI	1167	2.7711	2.0700	1.6923	0	8.6500
SIZE	1167	22.4414	22.0227	1.5258	20.0254	24.4866
LEV	1167	0.4669	0.4649	0.2154	0.0156	1.3518
LIQ	1167	1.8494	1.2290	2.8719	0.1668	68.9655
EQU	1167	36.8848	34.8700	15.0456	8.4480	89.9900
SOE	1167	0.5338	1	0.4991	0	1
AGE	1167	7.6026	7.6019	0.0033	7.5969	7.6094

### Correlation analysis

It can be seen from Table 3 that the correlation coefficient between enterprise environmental protection expenditure and financial performance is negatively correlated at the level of 1%, which preliminarily shows that enterprise environmental protection expenditure negatively affects the current performance of enterprises, but further proof is needed. The correlation coefficient between enterprise green technology innovation and financial performance is positively correlated at the 1% level. At the same time, the correlation coefficients between the variables are not high, which preliminarily shows that there is no serious multicollinearity problem between the variables.

**Table 3:** Coefficient matrix

	ROA	EPI	GTI	SIZE	LEV	LIQ	EQU	SOE	AGE
ROA	1								
EPI	− 0.110***	1							
GTI	0.308***	0.042	1						
SIZE	0.193***	− 0.125***	0.437***	1					
LEV	0.081***	0.111***	− 0.060**	− 0.004	1				
LIQ	− 0.085***	− 0.056*	− 0.013	− 0.016	− 0.484***	1			
EQU	− 0.051*	0.079***	− 0.067**	− 0.034	0.077***	− 0.056*	1		
SOE	0.081***	0.085***	− 0.080***	− 0.009	0.308***	− 0.106***	0.229***	1	
AGE	− 0.117***	− 0.032	0.104***	− 0.003	− 0.340***	0.156***	− 0.114***	− 0.541***	1

*** Significant at 1%; ** significant at 5%; * significant at 10%

In order to further detect whether there is multicollinearity among the independent variables, this study selects the variance inflation factor (VIF) to test the multicollinearity among the variables. The specific values are shown in Table 4. It can be seen from Table 4 that the variance inflation factor (VIF) of independent variables is in the range of 1.04–1.52, which is much smaller than 10, indicating that the independent variables pass the test and there is no multicollinearity problem among them.

**Table 4:** Multicollinearity analysis

Variable	VIF
EPI	1.04
GTI	1.26
SIZE	1.26
LEV	1.49
LIQ	1.31
EQU	1.06
SOE	1.52
AGE	1.50

### Analysis of regression results

Regression analysis is an analytical method used to study quantitative relationships among variables. In order to verify the research hypothesis proposed in this study, regression analysis was used to study the linear relationship between the dependent variable and the independent variables. Before performing regression analysis, this study uses *F* test and Hausman test to judge and screen mixed regression, fixed effect and random effect models. First, Model 1, Model 3, Model 5 and Model 6 were tested by *F* test. The test showed that the *p* values were all 0.000. The null hypothesis was rejected, and the fixed-effects model was considered better than mixed regression, and mixed regression was not accepted. Secondly, the Hausman test was performed on Model 1, Model 3, Model 5 and Model 6, and the *p* values were all 0.000. Therefore, the null hypothesis of “the random effect model is the correct model” was rejected, and the fixed effect model should be used. Therefore, the fixed effect model is used in this study to perform regression verification on the hypotheses H1a, H2a and H3a, and on this basis, a lag period is added to verify the lag effects of the hypotheses H1b, H2b and H3b.

#### Regression analysis of enterprise environmental protection expenditure and financial performance

The regression results of Model 1 in Table 5 show that the correlation coefficient between enterprise environmental protection expenditure and financial performance for the current period is − 0.0351, which is negatively correlated at the 5% significance level, indicating that there is a significant negative relationship between enterprise environmental protection expenditure and financial performance for the current period. Hypothesis H1a is proved. In addition, Model 2 shows the relationship between enterprise environmental protection expenditure and financial performance under the conditions of one lag period and two lag periods respectively. It can be found that the regression coefficient of environmental protection expenditure (EPI) is 0.1369 when it lags two periods, which is significant at the 1% level, and is most significant in the 3-year results. Therefore, the effect of enterprise environmental protection expenditure (EPI) on financial performance is significant. The effect is most significant at a two-period lag; hypothesis H1b is proven.

**Table 5:** Regression results of the impact of enterprise environmental protection expenditure on enterprise financial performance

Independent variables	Model 1	Model 2 Lag one period	Model 2 Lag two periods
EPI	− 0.0351**		
	(− 2.5264)		
EPI_i,t−1_		0.0480***	
		(4.7527)	
EPI_i,t−2_			0.1369***
			(6.9273)
SIZE	0.0014	0.0022**	0.0074***
	(1.3305)	(2.3086)	(6.1046)
LEV	0.0174	0.0414	0.0347
	(1.1827)	(1.3849)	(0.9285)
AGE	11.7161***	26.4910***	2.2573
	(2.9288)	(4.0944)	(0.6175)
EQU	− 0.0001	− 0.0003	− 0.0005
	(− 0.2769)	(− 0.4720)	(− 0.7981)
LIQ	0.0002	− 0.0015	− 0.0007
	(0.2727)	(− 1.4031)	(− 0.1455)
SOE	− 0.0087	− 0.0202	− 0.0132
	(− 0.8524)	(− 0.8129)	(− 0.5789)
YEAR	Yes	Yes	Yes
*N*	1167	662	426
adj. *R*^2^	0.468	0.356	0.530

*** Significant at 1%; ** significant at 5%; * significant at 10%

#### Regression analysis of enterprise green technology innovation and financial performance

The regression results of Model 3 in Table 6 show that the correlation coefficient between enterprise green technology innovation and financial performance in the current period is 0.0019, which is positively correlated at the 5% significance level, indicating that there is a significant relationship between enterprise green technology innovation and financial performance in the current period. Hypothesis H2a is validated. In addition, Model 4 shows the relationship between enterprise green technology innovation and financial performance under the conditions of one lag period and two lag periods respectively. It can be found that the regression coefficient of green technology innovation is 0.0151 when it lags two periods, which is significant at the 1% level, and is most significant in the 3-year results. Therefore, the impact of enterprise green technology innovation on financial performance has a time lag, and it is most significant when the lag is two periods. Hypothesis H2b is proved; it takes time to transform green technology innovation into financial performance and can bring higher profits.

**Table 6:** Regression results of the impact of enterprise green technology innovation on enterprise financial performance

Independent variables	Model 3	Model 4 Lag one period	Model 4 Lag two periods
GTI	0.0019**		
	(2.3141)		
GTI_i,t−1_		0.0011	
		(0.7637)	
GTI_i,t−2_			0.0151***
			(8.0552)
SIZE	0.0025**	0.0047***	− 0.0040
	(2.3391)	(2.8121)	(− 1.6099)
LEV	0.0164	0.0559*	0.0557
	(1.1116)	(1.7584)	(1.5596)
AGE	12.5720***	25.3105***	2.3810
	(3.2535)	(3.8759)	(0.4183)
EQU	− 0.0002	− 0.0001	− 0.0005
	(− 0.4282)	(− 0.2278)	(− 0.6047)
LIQ	0.0002	− 0.0010	0.0063
	(0.2598)	(− 0.8372)	(1.2673)
SOE	− 0.0082	− 0.0169	− 0.0074
	(− 0.7848)	(− 0.6271)	(− 0.3647)
YEAR	Yes	Yes	Yes
*N*	1167	662	426
adj. *R*^2^	0.442	0.226	0.445

*** Significant at 1%; ** significant at 5%; * significant at 10%

#### Regression analysis of synergetic effect of enterprise environmental protection expenditure and green technology innovation on financial performance

This study analyzes the synergetic effect of enterprise environmental protection expenditure and green technology innovation on financial performance in two steps. The first step is to incorporate the enterprise environmental protection expenditure and green technology innovation into the regression model; the second step is to add the interaction item EPI*GTI of enterprise environmental protection expenditure and green technology innovation on the basis of the first step. The results of the regression analysis can be seen in Table 7.

**Table 7:** Regression results of synergistic impact of enterprise environmental protection expenditure and green technology innovation on enterprise financial performance

Independent variables	Model 5	Model 6	Model 7 Lag one period	Model 8 Lag one period	Model 7 Lag two periods	Model 8 Lag two periods
EPI	− 0.0329**	− 0.0104***				
	(− 2.5340)	(− 5.6595)				
GTI	0.0023**	0.0314***				
	(2.1937)	(0.0979)				
EPI*GTI		0.3230***				
		(3.3880)				
EPI_i,t−1_			0.0479***	0.0811**		
			(4.6753)	(2.4479)		
GTI_i,t−1_			0.0001	0.0011		
			(0.0574)	(0.8544)		
EPI_i,t−1_*GTI_i,t−1_				− 0.0148		
				(− 1.5229)		
EPI_i,t−2_					0.1556***	0.1412***
					(10.3587)	(8.3201)
GTI_i,t−2_					0.0180***	0.0176***
					(16.8612)	(16.5264)
EPI_i,t−2_*GTI_i,t−2_						0.4923***
						(2.0220)
SIZE	0.0013	0.0014	0.0022	0.0023*	− 0.0078***	− 0.0076***
	(1.1991)	(1.2930)	(1.5773)	(1.6720)	(− 5.4941)	(− 5.4019)
LEV	0.0170	0.0192	0.0413	0.0405	− 0.0059	− 0.0033
	(1.1530)	(1.2915)	(1.3881)	(1.3761)	(− 0.2554)	(− 0.1391)
AGE	12.1887***	11.9571***	26.4892***	26.1923***	16.2053	15.5352
	(3.2179)	(2.9065)	(4.1014)	(4.1458)		
EQU	− 0.0001	0.0000	− 0.0003	− 0.0003	0.0001	0.0000
	(− 0.2933)	(0.0244)	(− 0.4705)	(− 0.4551)	(0.4177)	(0.1529)
LIQ	0.0002	0.0002	− 0.0015	− 0.0015	0.0015	0.0016
	(0.2776)	(0.2651)	(− 1.4003)	(− 1.4013)	(0.3258)	(0.3588)
SOE	− 0.0087	− 0.0096	− 0.0202	− 0.0201	− 0.0123	− 0.0125
	(− 0.8794)	(− 0.9060)	(− 0.8109)	(− 0.8299)	(− 0.7054)	(− 0.7171)
YEAR	Yes	Yes	Yes	Yes	Yes	Yes
*N*	1167	1167	662	662	426	426
adj. *R*^2^	0.469	0.501	0.355	0.380	0.772	0.773

*** Significant at 1%; ** significant at 5%; * significant at 10%

Model 5 is based on Model 1 and Model 3 by adding the independent variables of environmental protection expenditure (EPI) and green technology innovation (GTI) at the same time in this study, in order to examine the impact of another variable on enterprise financial performance by controlling one. After comparing Model 1, Model 3 and Model 5, it is found that when the independent variable environmental protection expenditure (EPI) is controlled, the regression coefficient of enterprise green technology innovation on financial performance becomes larger (0.0023 > 0.0019), indicating the adjustment effect of environmental protection expenditure. It enhances the positive impact of green technology innovation on the current financial performance of enterprises. Under the control of green technology innovation, the negative impact of environmental protection expenditure on the current financial performance of the company is reduced (0.0329 < 0.0351), indicating that the moderating effect of green technology innovation can reduce the negative impact of environmental protection expenditure on current financial performance to a certain extent.

In order to further illustrate the synergetic effect of enterprise environmental protection expenditure and green technology innovation on financial performance, the regression results of Model 6 and Model 8 are further compared. Model 6 adds the interaction item between enterprise environmental protection expenditure and green technology innovation (EPI*GTI) on the basis of Model 5, and Model 8 adds the lag period of independent variables and interaction item (EPI*GTI) on the basis of model 6. Model 6 shows that after adding the interaction term, not only the coefficient of the interaction term is significant at the level of 1%, but also the goodness of fit of the regression equation is improved compared with Model 5 (0.501 > 0.469), and the negative impact of enterprise environmental protection expenditure on the current financial performance is further weakened (0.0104 < 0.0329), and the significance level is improved; the positive impact of enterprise green technology innovation on current financial performance is further enhanced (0.0314 > 0.0023), and the significance level is improved. The hypothesis H3a is proved. Then, comparing the regression results of Model 6 and Model 8, the interaction term coefficient is greater when it lags behind two periods (0.4923 > 0.3230), which is significant at the level of 1%, and the goodness of fit of the regression equation is also the highest. It is assumed that H3b is proved.

### Robustness test

In this study, the variable substitution method is used to test the robustness. In order to study the robustness of the above test results, this paper uses the following method to test the robustness: Replacing the method of measuring the financial performance of the independent variable, using Tobin’s *Q* value (TQ) = the logarithm of the company’s market value/total assets as its substitute. On this basis, the regression equations of each model are repeated, and the results are shown in Table 8. Due to space limitations, for Model 2, Model 4, Model 6 and Model 8, only the results of two lags are shown. It can be seen from Table 8 that the coefficients of the main variables in each model and their positive and negative directions have not changed, and the significance level has not changed significantly, indicating that the regression results of each model have not changed significantly, and the null hypothesis is still established. In summary, the results of this study have good robustness.

**Table 8:** Robustness test results

Independent variables	Model 1	Model 2	Model 3	Model 4	Model 5	Model 6	Model 7	Model 8
EPI	− 0.0358**				− 0.0354**	− 0.0401		
	(− 2.3578)				(− 2.3940)	(− 0.8656)		
GTI			0.0045*		0.0044	0.0043		
			(0.8644)		(0.8387)	(0.7908)		
EPI*GTI						0.3127***		
						(0.1363)		
EPI_i,t−2_		0.1318**					0.1151**	0.1890***
		(2.1978)					(2.2007)	(3.2433)
GTI_i,t−2_				0.0183**			0.0161	0.0140***
				(1.8220)			(1.6245)	(1.3810)
EPI_i,t−2_								0.4203***
*GTI_i,t−2_								(2.2287)
SIZE	− 0.0090*	− 0.0036	− 0.0081	0.0128	− 0.0093*	− 0.0093*	0.0100	0.0091
	(− 1.7892)	(− 0.5776)	(− 1.5978)	(1.1061)	(− 1.8173)	(− 1.8154)	(0.8566)	(0.7722)
LEV	0.0527	− 0.2686	0.0513	− 0.1867	0.0519	0.0520	− 0.2323	− 0.2458
	(0.3943)	(− 1.0446)	(0.3807)	(− 0.7335)	(0.3855)	(0.3864)	(− 0.9011)	(− 0.9439)
AGE	53.1648	161.3606***	54.7161	138.6808***	54.3279	54.3133	148.9030***	152.3425***
	(0.5945)	(9.2355)	(0.6077)	(8.0818)	(0.6036)	(0.6032)	(7.8767)	(7.6588)
EQU	− 0.0019	− 0.0020	− 0.0020	− 0.0030	− 0.0019	− 0.0019	− 0.0025	− 0.0020
	(− 0.8790)	(− 0.5853)	(− 0.9126)	(− 0.8827)	(− 0.8870)	(− 0.8803)	(− 0.7712)	(− 0.6240)
LIQ	0.0052	0.0069	0.0052	0.0086	0.0052	0.0052	0.0050	0.0042
	(0.9873)	(0.2218)	(0.9860)	(0.2672)	(0.9864)	(0.9860)	(0.1579)	(0.1327)
SOE	− 0.1676	0.1740***	− 0.1670	0.1768***	− 0.1675	− 0.1676	0.1731***	0.1742***
	(− 0.9924)	(4.3932)	(− 0.9882)	(3.9385)	(− 0.9888)	(− 0.9887)	(3.9847)	(3.9912)
YEAR	Yes	Yes	Yes	Yes	Yes	Yes	Yes	Yes
*N*	1167	426	1167	426	1167	1167	426	426
adj. *R*^2^	0.402	0.596	0.401	0.597	0.401	0.401	0.601	0.602

*** Significant at 1%; ** significant at 5%; * significant at 10%

### Further analysis: heterogeneity analysis

#### Analysis on the heterogeneity of property rights

Due to the different nature of enterprise property rights, the synergetic effects of environmental protection expenditure and green technology innovation on enterprise financial performance may be different between state-owned enterprises and non-state-owned enterprises. Compared with non-state-owned enterprises, state-owned enterprises will be subject to more strict social supervision and pay more attention to social benefits. They are often inferior to non-state-owned enterprises in resource allocation efficiency and operation efficiency, but state-owned enterprises have significant advantages in organizational and institutional conditions and are easier to obtain bank loans tax incentives and other resources for environmental protection expenditure and green technology innovation (Wang et al. 2017; Cai et al. 2020). Therefore, it is necessary to analyze whether the synergetic effect of environmental protection expenditure and green technology innovation on enterprise financial performance will change with the change of the nature of enterprise property rights. The sample is divided into state-owned enterprises and non-state-owned enterprises. The regression analysis results of model 6 and model 8 are shown in Table 9. Due to space constraints, Table 9 only shows the regression results of model 8 lagging behind two periods. It can be seen from Table 9 that compared with non-state-owned enterprises, the environmental protection expenditure of state-owned enterprises has less negative effect on the current financial performance of enterprises, and the green technology innovation has a greater effect on the current financial performance of enterprises (0.0017 >  − 0.0007), and the environmental protection expenditure lagging behind two periods has a greater positive effect on the financial performance of enterprises (0.1418 > 0.1334). The interaction coefficient of environmental protection expenditure and green technology innovation of state-owned enterprises is significant in the current period and lag period, and the coefficient of lag period is larger (0.4817 > 0.3112), and the goodness of fit of regression equation is higher. In non-state-owned enterprises, the interaction coefficient is not significant in the current period and the lag period. The possible reason is that state-owned enterprises are subject to stricter supervision, have stronger initiative in environmental protection expenditure and green technology innovation and are easier to obtain tax incentives and bank loans for environmental protection project construction and green technology innovation. Therefore, the synergetic promotion effect of enterprise environmental protection expenditure and green technology innovation on financial performance is differentiated in enterprises with different property rights, and the synergetic promotion effect is more obvious in state-owned enterprises.

**Table 9:** Analysis of differences in the nature of property rights

Independent variables	Property rights			
	State-owned enterprises		Non-state-owned enterprises	
	Model 6	Model 8	Model 6	Model 8
EPI	− 0.1071***		− 0.1651**	
	(− 5.1147)		(− 2.3999)	
GTI	0.0017		− 0.0007	
	(1.2069)		(− 0.5798)	
EPI*GTI	0.3112***		0.0371	
	(3.0576)		(1.4751)	
EPI_i,t−2_		0.1418***		0.1334***
		(7.2851)		(3.6119)
GTI_i,t−2_		0.0178***		0.0187***
		(11.6346)		(10.5864)
EPI_i,t−2_		0.4817***		− 0.0050
*GTI_i,t−2_		(1.7233)		(− 0.2211)
SIZE	0.0030	− 0.0051***	0.0001	− 0.0104***
	(1.6139)	(− 2.6999)	(0.1224)	(− 4.2612)
LEV	0.0174	− 0.0104	0.0152	− 0.0083
	(0.7812)	(− 0.3150)	(0.6818)	(− 0.2218)
AGE	12.6119***	20.5830	14.5910	
	(3.0004)			
EQU	− 0.0003	0.0000	0.0004	0.0005
	(− 0.5690)	(0.0337)	(0.6978)	(0.6526)
LIQ	0.0007**	0.0080	− 0.0016	− 0.0005
	(2.3042)	(1.5648)	(− 0.7096)	(− 0.0923)
YEAR	Yes	Yes	Yes	Yes
*N*	623	252	544	174
adj. *R*^2^	0.487	0.795	0.533	0.748

*** Significant at 1%; ** significant at 5%; * significant at 10%

#### Analysis on the heterogeneity of corporate governance

Corporate governance is the general name of the mechanism to solve various problems caused by the separation of ownership and control in modern corporate system. The level of corporate governance directly reflects the level of effective operation of corporate governance mechanism. Corporate governance can be divided into broad sense and narrow sense. The broad sense of corporate governance refers to the relationship between the company and all stakeholders through internal and external systems, while the narrow sense of corporate governance refers to the internal governance structure composed of the board of directors, the board of supervisors, the general meeting of shareholders and the management (Ciftci et al. 2019; Domadenik et al. 2016). The internal governance structure of the company plays an important role in the environmental protection expenditure and green technology innovation of enterprises (Ma et al. 2021). This study focuses on the impact of corporate governance in a narrow sense on the environmental protection expenditure and green technology innovation of enterprises. Enterprises’ increasing environmental protection expenditure and green technology innovation are often subject to a series of institutional arrangements directly related to corporate governance. Enterprises with different levels of corporate governance have slightly different attitudes and policies towards environmental protection expenditure and green technology innovation, which may have a differentiated impact on enterprise financial performance. Therefore, it is of great significance to study the synergetic effect of enterprise environmental protection expenditure and green technology innovation on financial performance under the difference of corporate governance level. This paper uses the principal component analysis method to construct comprehensive indicators from the three aspects of supervision, incentive and decision-making to measure the level of corporate governance, as shown in Table 10. Then, based on the seven indicators in Table 10, the principal component analysis method is used to construct the corporate governance index. The first principal component obtained from principal component analysis is used as a comprehensive index to reflect the level of corporate governance. The higher the score, the better the level of corporate governance.

**Table 10:** Index structure of corporate governance level

		Variable symbol	Variable description
Supervision mechanism	Board supervision	Outratio	Number of independent directors/number of directors
		Board	Number of directors
	Equity structure supervision	Inst_Share	Total shareholding ratio of institutions/100
		Share_Balance	Sum of shareholding ratio of the second to fifth largest shareholders/shareholding ratio of controlling shareholders
Incentives		Mana_Pay	Total annual salary of directors, supervisors and senior executives
		Mana_Share	Number of shares held by the management/paid-in capital or capital stock
Decision-making mechanism		Dual	1 if the chairman and the general manager serve concurrently, otherwise 0

The corporate governance level is calculated according to the above standards, and on this basis, the grouping regression of Model 6 and Model 8 is carried out. Finally, Table 11 shows the results of Model 8 lagging behind two periods. The results show that the synergetic promotion effect of environmental protection expenditure and green technology innovation on enterprise financial performance in heavy polluting industries with high corporate governance level is significant at the level of 1% in the independent variables current period and lag two periods, and the interactive term coefficient is higher in lag two periods (0.4801 > 0.3293), and the goodness of fit of regression equation is greater. However, the synergetic effect of environmental protection expenditure and green technology innovation on enterprise financial performance in heavy polluting industries with low corporate governance level is not significant. The possible reason is that the internal interests of enterprises with low governance level are uncoordinated, pay attention to the growth of short-term financial performance, are unwilling to carry out environmental protection expenditure and green technology innovation and ignore the long-term interests of enterprises. Companies with a high level of corporate governance can more effectively resolve internal differences, pay attention to safeguarding the interests of stakeholders and maintain good relations with stakeholders, so as to reduce unnecessary costs consumed by various stakeholders and pay more attention to the long-term development of enterprises. Therefore, it will stimulate the “first mover advantage” of enterprises through environmental protection expenditure and green technology innovation, develop new green products, establish the “green” image of enterprises, maintain friendly relations with stakeholders and improve the market competitiveness of enterprises.

**Table 11:** Analysis on the difference of corporate governance level

Independent variables	Corporate governance level			
	High level		Low level	
	Model 6	Model 8	Model 6	Model 8
EPI	− 0.1099***		0.0502	
	(− 4.9150)		(0.5334)	
GTI	0.0001		0.0011	
	(0.0583)		(0.6888)	
EPI*GTI	0.3293***		− 0.0608	
	(3.1758)		(− 1.4078)	
EPI_i,t−2_		0.1094***		0.1392
		(4.2921)		(1.3084)
GTI_i,t−2_		0.0184***		0.0180***
		(10.5101)		(9.9035)
EPI_i,t−2_		0.4801***		0.0130
_*_GTI_i,t−2_		(2.1469)		(0.4344)
SIZE	0.0025	− 0.0082***	− 0.0012	− 0.0109***
	(1.1808)	(− 3.4129)	(− 1.1103)	(− 4.4762)
LEV	0.0623	0.0563	− 0.0025	0.0221
	(1.6144)	(1.3835)	(− 0.1229)	(0.3804)
AGE	16.1308***	− 0.1468	43.2260***	
	(3.1519)	(− 0.0084)	(3.7023)	
EQU	− 0.0004	− 0.0004	0.0007	0.0018**
	(− 0.7688)	(− 0.3646)	(1.4224)	(2.2900)
LIQ	− 0.0062**	0.0146	0.0020	− 0.0034
	(− 2.5589)	(1.5751)	(0.9260)	(− 0.5933)
SOE	− 0.0373***	− 0.0343***	− 0.0258***	
	(− 5.3212)	(− 4.6392)	(− 4.3395)	
YEAR	Yes	Yes	Yes	Yes
*N*	551	209	550	180
adj. *R*^2^	0.467	0.827	0.571	0.715

*** Significant at 1%; ** significant at 5%; * significant at 10%

## Conclusions and implications

Through the fixed effect test and lag effect test of the panel data of Listed Companies in China’s heavy polluting industry, it is found that the environmental protection expenditure of enterprises in heavy polluting industry is negatively correlated with the current financial performance, and has a lag effect on the positive impact on the financial performance, which is the most significant in the two lag periods; the green technology innovation of enterprises in heavy polluting industry is positively correlated with the current financial performance, and the positive impact is the most significant when it lags behind two periods; the environmental protection expenditure and green technology innovation of enterprises in heavy polluting industries regulate each other and promote the current financial performance of enterprises, and the synergetic effect is the most significant when it lags behind two periods. On this basis, the heterogeneity analysis shows that: compared with non-state-owned enterprises, the synergetic effect of environmental protection expenditure and green technology innovation on enterprise financial performance is more significant in state-owned enterprises; compared with companies with low corporate governance level, the synergetic effect of environmental protection expenditure and green technology innovation on corporate financial performance is more significant in enterprises with high corporate governance level.

According to the above conclusions, this study puts forward the following three suggestions. First, enterprises in heavy polluting industries should integrate environmental protection expenditure and green technology innovation. This study proves that enterprises’ environmental protection expenditure and green technology innovation alone cannot maximize the interests of enterprises. They must include increasing environmental protection expenditure and green technology innovation into the enterprise strategy at the same time, and there is a certain lag in the synergetic promotion effect of environmental protection expenditure and green technology innovation on enterprise financial performance. Enterprises should take a long-term view; capital investment should not be interrupted because the increase of environmental protection expenditure and green technology innovation have a weak improvement on the current financial performance of enterprises. Increasing environmental protection expenditure and carrying out green technology innovation are not only conducive to the short-term development of enterprises, but also conducive to safeguarding the long-term interests of enterprises.

Second, the government should give more preferential policies and support to non-state-owned enterprises. This study finds that the synergetic promotion effect of environmental protection expenditure and green technology innovation on enterprise financial performance is not significant in non-state-owned enterprises. Therefore, in the future, the government should focus on non-state-owned enterprises and give them policy support so that they have sufficient funds for environmental protection expenditure and green technology innovation, so as to realize the transformation and upgrading of enterprises.

Finally, enterprises in heavy polluting industries should strengthen internal governance and improve the level of corporate governance. This study confirms that the level of corporate governance can affect the synergetic promotion of enterprise environmental protection expenditure and green technology innovation on enterprise financial performance. In companies with high corporate governance level, enterprises can better deal with internal interest contradictions, pay attention to maintaining the relationship with various stakeholders and realize the long-term development of enterprises. Therefore, enterprises should strengthen the construction of corporate governance and create a good corporate governance environment for enterprise decision-making and development.

## Data Availability

All data generated or analyzed during this study were included in this published article. In addition, the datasets used or analyzed during the current study were available from the corresponding author on reasonable request.
